# Apparent diffusion coefficient histogram shape analysis for monitoring early response in patients with advanced cervical cancers undergoing concurrent chemo-radiotherapy

**DOI:** 10.1186/s13014-016-0715-6

**Published:** 2016-10-22

**Authors:** Jie Meng, Lijing Zhu, Li Zhu, Huanhuan Wang, Song Liu, Jing Yan, Baorui Liu, Yue Guan, Yun Ge, Jian He, Zhengyang Zhou, Xiaofeng Yang

**Affiliations:** 1 Department of Radiology, Nanjing Drum Tower Hospital, The Affiliated Hospital of Nanjing University Medical School, Nanjing, China 210008; 2 The Comprehensive Cancer Centre of Drum Tower Hospital, The Affiliated Hospital of Nanjing University Medical School, Nanjing, China 210008; 3 School of Electronic Science and Engineering, Nanjing University, Nanjing, China 210046; 4 Department of Radiation Oncology and Winship Cancer Institute, Emory University, Atlanta, GA 30322 USA

**Keywords:** Cervical cancer, Treatment response, Diffusion-weighted magnetic resonance imaging, Apparent diffusion coefficient, Histogram shape

## Abstract

**Background:**

To explore the role of apparent diffusion coefficient (ADC) histogram shape related parameters in early assessment of treatment response during the concurrent chemo-radiotherapy (CCRT) course of advanced cervical cancers.

**Methods:**

This prospective study was approved by the local ethics committee and informed consent was obtained from all patients. Thirty-two patients with advanced cervical squamous cell carcinomas underwent diffusion weighted magnetic resonance imaging (b values, 0 and 800 s/mm^2^) before CCRT, at the end of 2nd and 4th week during CCRT and immediately after CCRT completion. Whole lesion ADC histogram analysis generated several histogram shape related parameters including skewness, kurtosis, s-sD_av_, width, standard deviation, as well as first-order entropy and second-order entropies. The averaged ADC histograms of 32 patients were generated to visually observe dynamic changes of the histogram shape following CCRT.

**Results:**

All parameters except width and standard deviation showed significant changes during CCRT (all *P* < 0.05), and their variation trends fell into four different patterns. Skewness and kurtosis both showed high early decline rate (43.10 %, 48.29 %) at the end of 2nd week of CCRT. All entropies kept decreasing significantly since 2 weeks after CCRT initiated. The shape of averaged ADC histogram also changed obviously following CCRT.

**Conclusions:**

ADC histogram shape analysis held the potential in monitoring early tumor response in patients with advanced cervical cancers undergoing CCRT.

## Background

Nowadays, concurrent chemo-radiotherapy (CCRT) remains the standard treatment protocol for advanced cervical cancers [1]. How to monitor tumor response to CCRT early and accurately turns out to be a major challenge in the era of personalized medicine. The conventional method to assess tumor response is to observe the changes in tumor size using computed tomography (CT) or magnetic resonance (MR) imaging. However, those morphologic alterations significantly lag behind the biological and molecular changes that occur early in responders [2]. Diffusion weighted (DW) imaging can noninvasively reflect the mobility of water molecules in vivo. Apparent diffusion coefficient (ADC) histogram analysis can evaluate microstructural heterogeneity within the whole tumor [3]. Several studies have shown that ADC histogram analysis was useful in characterizing cervical cancer and normal cervix, distinguishing between well or moderately and poorly differentiated cervical cancers and identifying squamous cell carcinoma from adenocarcinoma of the cervix [4–7].

Histogram shape related parameters refer to the indexes which can reflect the shape and general features of the histogram distribution, including skewness, kurtosis, s-sD_av_, width, standard deviation as well as entropy. Unlike point-specific parameters such as percentiles or ADC_min_ reflecting only a specific portion of the tumor, histogram shape related parameters take the advantage of showing characteristics of the entire tumor [8]. The administration of effective non-surgical anti-cancer therapy results in a series of pathophysiological reactions involving necrosis, apoptosis and tumor lysis, the value and distribution of ADC parameters within the tumor will change as well [9]. Therefore, we hypothesized that the histogram shape related parameters could be used to monitor tumor response to anti-cancer therapies. Numerous studies have proved that skewness and kurtosis were associated with treatment efficacy in various tumors such as ovarian cancer [10], head and neck squamous carcinoma (HNSCC) [11] and glioma [12]. But there were few reports on the role of s-sDav, width and standard deviation. In addition, as a new indicator, entropy has been increasingly studied on tumor prognosis in recent years, and several studies have proved it as one of prognostic factors of tumors such as prostate cancers and osteosarcomas [13, 14].

Generally speaking, cell morphologic and microstructural heterogeneity of cervical cancer are greater than normal cervix. Shape of the histogram of cervical cancer was significantly different from that of normal cervix [5]. To our knowledge, there were no reports investigating changes of ADC histogram shape for monitoring cervical cancer response to CCRT. So, the purpose of this study was to explore how histogram shape related parameters and the shape of averaged ADC histogram changed during the CCRT course of advanced cervical cancer and whether those changes could serve as early biomarkers for therapeutic response.

## Methods

### Patients

This study was approved by the ethics committee of the Institutional Review Board of Nanjing Drum Tower Hospital (reference number: 20140116) and written informed consents were obtained from all the patients. Between October 2014 and June 2015, thirty-two patients (mean age, 52 years; range, 24–76 years) were recruited to this prospective study. The inclusion criteria consisted of: (a) women aged over 18 years with biopsy-proven cervical cancer and clinically diagnosed as advanced cervical cancer (staged IIB to IVA based on the International Federation of Gynecology and Obstetrics (FIGO) classification), (b) no previous treatment for cervical cancer before the first MR examination, (c) being scheduled to receive CCRT in our hospital, (d) finishing the follow-up MR examinations on time.

### Concurrent chemo-radiotherapy

All the patients were scheduled to undergo radiotherapy (RT) in combination with concurrent nedaplatin-containing chemotherapy. RT consisted of external beam radiation therapy (EBRT) and intracavitary brachytherapy (ICBT). EBRT was delivered to the whole pelvis at 1.8-2.0 Gy daily, 5 days a week, with a total dose of 45–50 Gy. ﻿From the last week of EBRT, ﻿﻿ICBT was given twice a week with a fraction dose of 5 Gy to point A (2 cm above the distal end of the lowest cervix and 2 cm lateral to the midline), and the total dose was 30–40 Gy. The total radiation time was ﻿within 8 weeks. All the patients received chemotherapy (﻿six cycles ﻿of weekly nedaplatin or four cycles of bi-weekly nedaplatin plus paclitaxel/docetaxel) combined with EBRT.

### MR imaging protocol

All the patients received MR examinations at four time points: before CCRT, at the end of 2nd and 4th week during CCRT and immediately after CCRT completion. All the examinations were performed with a 3.0 T MR scanner (Ingenia 3.0 T, Philips Healthcare, Best, the Netherlands) with a 16-channel torso phased-array body coil. Patients were asked to take clyster 2–3 h before the MR examination in order to reduce artifacts induced by gas and feces within the rectum. Axial DW imaging was performed with a non-breath-hold spin-echo echo-planar-imaging sequence (b value, 0 and 800 s/mm^2^; repetition time/echo time, 3523–6000 ms/shortest ms; slice thickness/gap, 4 mm/1 mm; matrix size, 132 × 157; field of view, 24 × 24 cm; number of slices: 24; number of signal averaged, 2). Besides, axial T1-weighted high resolution isotropic volume examination sequence, axial and sagittal T2-weighted turbo spin-echo sequences, axial and sagittal T2-weighted spectral attenuated inversion recovery sequences, axial and sagittal contrast enhancerd-T1 high resolution isotropic volume examination sequences were also acquired. The total MR examination cost about thirty minutes. All the situations and MR parameters were kept exactly the same during the whole follow-up process.

### Post processing and ADC histogram shape related parameters acquisition

The DW images were loaded into a workstation (Extended MR Workspace 2.6.3.4; Philips Medical Systems, Best, the Netherlands) and ADC maps were generated automatically using the mono-exponential model. Two radiologists (XX and XX) with 3 and 7 years’ experience in gynecological imaging performed the histogram analysis, and the workflow is described as follows:

1. DW images and the corresponding ADC maps were imported into our in-house software (Image Analyzer 1.0, China).
2. The two radiologists were informed of the diagnosis and clinical treatment information, and they together manually drew regions of interest (ROIs) slice by slice on the DW images (b = 800 s/mm^2^) referring to other sequences successively in accordance with the order of follow-up. Each ROI covered the edge of the lesion on each slice avoiding obvious artifacts. At time point 4, given a complete remission, we measured five identical round ROIs (each 5 mm^2^) in the former tumor region (not including any peri-cervical tissue) at time point 3. Similarly, if there was no obvious residual lesion at time points 3 and 4, we measured several round ROIs in the former tumor region at time point 2. The outlines of ROIs drawn on each slice would be automatically copied to the exact same location of the corresponding ADC maps in real time.
3. After selecting all the ROIs that covered the entire volume of the lesion, a button was clicked in our software, and a set of histogram shape related parameters as well as tumor volume were generated automatically including: (a) skewness, a measure of the asymmetry of the ADC value distribution around its mean; (b) kurtosis, a measure of how peaked a histogram is; (c) s-sD_av_, width of the ADC histogram corresponding to half of the histogram peak; (d) width, width between the 10th and 90th percentile of the ADC histogram; (e) standard deviation, the square root of the variance of all ADC values within VOI; (f) entropy, a measure of the randomness of ADC value distribution in an ADC histogram.

There were two types of entropy calculated with our software, first-order entropy and second-order entropies. The definition and formulas of the two types of entropy are described as follows:

First-order entropy describes the distribution variation of grey levels over the VOI, which was calculated with the following formula:

$$ \mathrm{entropy}=-{\displaystyle \sum_{i=0}^{G-1}{p}_i \log \left({p}_i\right)} $$

G is the number of gray levels within the VOI. *p*_*i*_ represents the probability of grey level *i* across the VOI and is computed by dividing the number of the grey level *i* by the total pixel number within the VOI. The larger the gray levels’ variation is, the greater the first-order entropy will be.

Second-order entropy represents the frequency of a pair of pixels with a certain distance in a certain direction occurring in the image, and can provide the spatial information of ADC distribution. Second-order entropy was calculated with the following formula:

$$ \mathrm{entropy}\left(\mathrm{H}\right)=-{\displaystyle \sum_{i=0}^{G-1}{\displaystyle \sum_{j=0}^{G-1}p\left(i,j\right)}} \log \left(p\left(i,j\right)\right) $$

G is the number of gray levels within the VOI. *p*(*i*, *j*) represents the probability of a pair of pixels with grey levels *i* and *j* occurs in the original image. And those two pixels are spatially dependent in the original image. Our in-house software calculated 12 s-order entropies deriving from different directions (entropy(H)_1–12_), as well as the averaged value of the 12 s-order entropies, namely entropy(H)_mean_.

### Generation of the averaged ADC histogram

In order to visually observe dynamic changes of the ADC histogram shape, we used the software (Matlab, R2010b; Mathworks, Natick, Mass) to generate the averaged ADC histogram and its fitting curve of each time point. The workflow is described as follows: for each time point, all ADC values of each patient were divided into a number of isometric intervals with a bin size of 50 × 10^−6^ mm^2^/s. Then we calculated the averaged frequencies in the same interval of the 32 patients. All the ADC intervals and their corresponding averaged frequencies were imported into the software to generate the averaged ADC histogram as well as the fitting curve.

### Treatment outcome evaluation

Response to CCRT was determined by the shrinkage of tumor size. The longest diameter of the tumor was measured on a specific slice of axial T2-weighted images with the largest tumor section. According to Response Evaluation Criteria in Solid Tumors (RECIST) [15], complete response (CR) was concluded if there was no residual tumor; partial response (PR) was concluded if the longest diameter of the tumor was less than 70 % of the original size; progressive disease (PD) was concluded if there was at least a 20 % increase in the longest diameter of tumor in comparison with the original size; stable disease (SD) was concluded if there was neither sufficient shrinkage to qualify for PR nor sufficient increase to qualify for PD. After CCRT, all the patients in this study achieved efficient local control. The patients and treatment characteristics are shown in Table 1.

**Table 1:** Patients of cervical cancers and treatment characteristics

Clinical features	Values
No. of patients	32
Mean age{range}	52 years {24–76}
FIGO stage	
II	18 (56.3 %)
III	9 (28.1 %)
IV	5 (15.6 %)
Histological type	
Squamous cell carcinoma	32 (100 %)
Adenocarcinoma	0
Treatment outcome	
Complete response	27 (84.4 %)
Partial response	5 (15.6 %)

Note: Numbers in parentheses are percentages

FIGO = the International Federation of Gynecology and Obstetrics

### Statistical analysis

Statistical analysis was performed with SPSS 13.0 software (SPSS Inc., Chicago, IL). Changes of the histogram shape related parameters with time were tested using variance analysis of repeated measurements. Least significant difference method was adopted for further comparison between parameters at each two time points. All the *P* values were two-tailed, and *P* values less than 0.05 were considered statistically significant.

## Results

Tables 2 and 3 list the mean values of ADC histogram shape related parameters at four time points. The variation trends of those parameters fell into four patterns: (i) rapid descending type; (ii) platform-descending type; (iii) platform-rising type; (iv) platform type.

**Table 2:** ADC histogram shape related parameters during the CCRT course in patients with advanced cervical cancers

Parameter	Time point 1	Time point 2	Time point 3	Time point 4
Skewness	1.16 ± 0.53	0.66 ± 0.57*	0.17 ± 0.30^**§**^	0.04 ± 0.16
Kurtosis	2.34 ± 1.56	1.21 ± 1.16*	0.30 ± 0.51^**§**^	0.11 ± 0.10
First-order entropy	6.22 ± 0.45	5.74 ± 0.89	4.17 ± 1.40^**§**^	3.38 ± 1.19^**#**^
Entropy(H)_mean_	10.22 ± 2.50	9.69 ± 2.36	6.93 ± 2.43^**§**^	5.55 ± 1.88^**#**^
s-sD_av_ (×10^−6^ mm^2^/s)	555.43 ± 453.93	623.00 ± 419.74	801.11 ± 591.85	1083.86 ± 560.68^**#**^
Width (×10^−6^ mm^2^/s)	705.43 ± 276.28	648.39 ± 249.47	714.68 ± 353.48	649.79 ± 523.89
Standard deviation	294.68 ± 94.37	271.56 ± 91.24	277.81 ± 134.01	270.68 ± 132.35
Longest diameter (cm)	4.49 ± 1.09	3.19 ± 1.13*	1.55 ± 1.40^**§**^	0.30 ± 0.69^**#**^
Volume (cm^3^)	30.95 ± 23.97	15.74 ± 16.46*	3.11 ± 7.65^**§**^	0.34 ± 0.79

Note: *ADC* apparent diffusion coefficient, *CCRT* concurrent chemo-radiotherapy

Data are presented as mean ± standard deviation

* *P* < 0.05: data show statistical difference between time point 2 and 1; ^**§**^ *P* < 0.05: data show statistical difference between time point 3 and 2; ^**#**^ *P* < 0.05: data show statistical difference between time point 4 and 3

Time point 1: before CCRT; Time point 2: at the end of 2nd week of CCRT; Time point 3: at the end of 4th week of CCRT; Time point 4: immediately after CCRT completion

**Table 3:** Second-order entropies during the CCRT course in patients with advanced cervical cancers

Second-order entropy	Time point 1	Time point 2	Time point 3	Time point 4
entropy(H)_1_	10.24 ± 2.53	9.15 ± 2.89	5.06 ± 3.49^**§**^	3.12 ± 2.72^**#**^
entropy(H)_2_	10.27 ± 2.53	9.18 ± 2.89	5.10 ± 3.53^**§**^	3.22 ± 2.74^**#**^
entropy(H)_3_	10.25 ± 2.53	9.14 ± 2.96	4.96 ± 3.63^**§**^	3.08 ± 2.78^**#**^
entropy(H)_4_	10.27 ± 2.49	9.18 ± 2.85	5.16 ± 3.41^**§**^	3.28 ± 2.59^**#**^
entropy(H)_5_	10.28 ± 2.50	9.21 ± 2.87	5.20 ± 3.42^**§**^	3.38 ± 2.62^**#**^
entropy(H)_6_	10.25 ± 2.52	9.16 ± 2.94	5.09 ± 3.47^**§**^	3.25 ± 2.66^**#**^
entropy(H)_7_	10.24 ± 2.50	9.13 ± 2.87	4.96 ± 3.43^**§**^	3.09 ± 2.55^**#**^
entropy(H)_8_	10.25 ± 2.51	9.15 ± 2.90	5.09 ± 3.34^**§**^	3.32 ± 2.52^**#**^
entropy(H)_9_	10.22 ± 2.53	9.11 ± 2.98	4.88 ± 3.55^**§**^	3.19 ± 2.57^**#**^
entropy(H)_10_	10.45 ± 2.42	9.98 ± 2.05	6.66 ± 2.66^**§**^	5.07 ± 2.28^**#**^
entropy(H)_11_	10.18 ± 2.61	9.67 ± 2.35	6.86 ± 2.53^**§**^	5.40 ± 2.07^**#**^
entropy(H)_12_	10.45 ± 2.43	9.98 ± 2.06	6.65 ± 2.71^**§**^	5.19 ± 2.19^**#**^

Note: *ADC* apparent diffusion coefficient, *CCRT* concurrent chemo-radiotherapy

Data are presented as mean ± standard deviation

^**§**^ *P* < 0.05: data show statistical difference between time point 3 and 2; ^**#**^ *P* < 0.05: data show statistical difference between time point 4 and 3

Time point 1: before CCRT; Time point 2: at the end of 2nd week of CCRT; Time point 3: at the end of 4th week of CCRT; Time point 4: immediately after CCRT completion

**Fig. 1:**
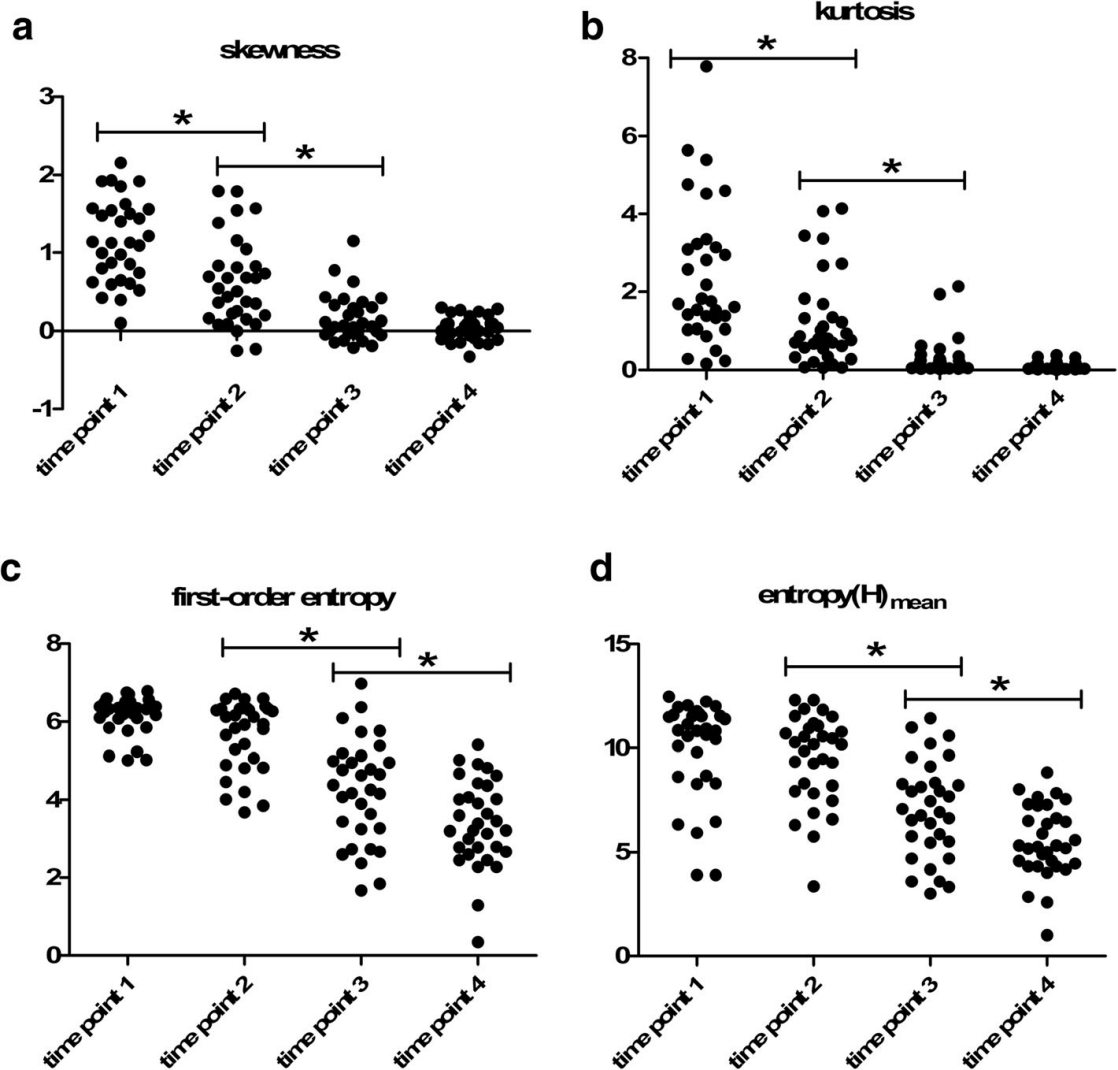
The variation trends of four kinds of ADC histogram parameters during concurrent chemo-radiotherapy (CCRT) in patients with cervical cancers. **a**, **b** Skewness and kurtosis show a quick and significant decrease from pre-CCRT to the end of 4th week of CCRT. **c**, **d** First-order entropy and entropy(H)_mean_ show no significant early change followed by a progressive and remarkable decrease after 2nd week of CCRT. *: *P* < 0.05. time point 1: before CCRT; time point 2: at the end of 2nd week of CCRT; time point 3: at the end of 4th week of CCRT; time point 4: immediately after CCRT completion
(ii) The platform-descending type, including first-order entropy and all second-order entropies, showed no significant early change followed by a progressive and remarkable decrease after time point 2 (Fig. 1c, d).
(iii) The platform-rising type, involving s-sD_av_, remained stable followed by a significant increase after time point 3.
(iv) The platform type, including width and standard deviation, remained stable during the whole course of CCRT.

(i) The rapid descending type, including skewness and kurtosis, showed quick and significant decrease from pre-CCRT to the end of 4th week of CCRT (Fig. 1a, b). The early decline rate of skewness and kurtosis were 43.10 % and 48.29 % at time point 2, respectively.

**Fig. 2:**
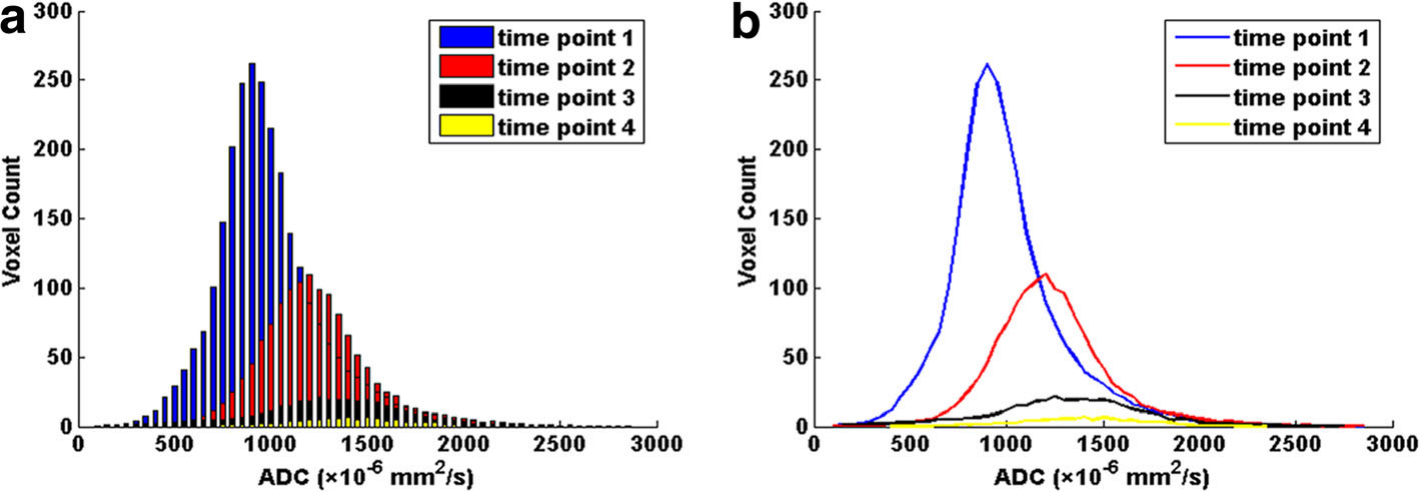
Dynamic changes of the averaged apparent diffusion coefficient (ADC) histogram and the corresponding histogram curve of 32 patients with advanced cervical cancers during concurrent chemo-radiotherapy (CCRT) (with a bin size of 50 × 10^−6^ mm^2^/s). **a** The averaged ADC histogram changes continuously during the course of CCRT. **b** The corresponding histogram curve gradually moves toward the right and turns into a more symmetrical shape with conspicuous descending peak as treatment continues. time point 1: before CCRT; time point 2: at the end of 2nd week of CCRT; time point 3: at the end of 4th week of CCRT; time point 4: immediately after CCRT completion

As treatment continued, the averaged ADC histogram gradually moved toward the right and turned into a more symmetrical shape with conspicuous descending peak (Fig. 2).

The mean values of tumor longest diameter and volume are also shown in Table 2. We found that both tumor size and volume showed a decreasing trend throughout CCRT, which was consistent with that of skewness, kurtosis and entropies (Tables 4 and 5). In addition, we failed to detect any significant difference of any ADC histogram shape related parameters of healthy cervical tissue between any two time points (Table 6).

**Table 4:** Correlations between the change of tumor size and ADC parameters during CCRT in patients with cervical cancers

Δ Parameter		Correlation coefficient	*P*
Change of tumor size	skewness	0.632	0.006*
	kurtosis	0.760	<0.001*
	s-sD_av_	0.012	0.963
	width	−0.110	0.673
	standard deviation	−0.074	0.779
	first-order entropy	0.664	0.004*
	entropy(H)_mean_	0.642	0.005*

Note: * *P* < 0.05. Δ Parameter = parameter values at time point 4 – parameter values at time point 1

**Table 5:** Correlations between the change of tumor volume and ADC parameters during CCRT in patients with cervical cancers

Δ Parameter		Correlation coefficient	*P*
Change of tumor volume	skewness	0.872	<0.001*
	kurtosis	0.947	**<**0.001*
	s-sDav	−0.234	0.231
	width	−0.309	0.109
	standard deviation	−0.203	0.300
	first-order entropy	0.147	0.456
	entropy(H)_mean_	−0.055	0.782

Note: *, *P* < 0.05. Δ Parameter = parameter values at time point 4 – parameter values at time point 1

**Table 6:** ADC histogram shape related parameters of healthy cervical tissue during CCRT in patients with cervical cancers

Parameter	Time point 1	Time point 2	Time point 3	Time point 4
Skewness	0.03 ± 0.12	0.01 ± 0.15	0.04 ± 0.11	0.02 ± 0.13
Kurtosis	0.09 ± 0.03	0.07 ± 0.03	0.07 ± 0.04	0.07 ± 0.02
First-order entropy	3.75 ± 0.38	3.52 ± 0.36	3.48 ± 0.16	3.73 ± 0.27
Entropy(H)_mean_	6.16 ± 0.56	5.71 ± 0.60	5.64 ± 0.26	5.96 ± 0.67
s-sD_av_ (×10^−6^ mm^2^/s)	1114.40 ± 602.64	1190.40 ± 427.14	1200.70 ± 451.39	1187.00 ± 484.02
Width (×10^−6^ mm^2^/s)	769.90 ± 227.77	746.10 ± 281.49	782.80 ± 316.62	739.10 ± 154.28
Standard deviation	283.47 ± 81.58	286.46 ± 89.72	292.76 ± 119.15	286.21 ± 56.61

Note: ADC, apparent diffusion coefficient; CCRT, concurrent chemo-radiotherapy. Data are presented as mean ± standard deviation. Time point 1: before CCRT; Time point 2: at the end of 2nd week of CCRT; Time point 3: at the end of 4th week of CCRT; Time point 4: immediately after CCRT completion

## Discussion

In this study, we investigated dynamic changes of ADC histogram shape related parameters following CCRT in patients with advanced cervical squamous cell carcinomas, and found that tumor response (CR or PR) was associated with decrease of skewness, kurtosis and entropy during CCRT. Those changes reflected on the averaged ADC histogram as a shift toward the right and adoption of a more symmetrical shape with conspicuous descending peak.

Our study showed that skewness decreased rapidly as early as 2 weeks after CCRT initiated. King et al.’s study on HNSCC also revealed that skewness decreased significantly within 2 weeks after chemotherapy [11]. However, some other studies reported slightly different results. Kyriazi et al.’s study on ovarian cancer and Tyagi et al.’s study on oropharyngeal squamous carcinoma with metastatic lymph nodes both found that skewness first increased and then decreased at the later stage of therapy [10, 16]. Theoretically, a positive skewness means a histogram curve with a big left shoulder, suggesting a large portion of highly cellular component, while a negative skewness means a histogram curve with a big right shoulder, suggesting a substantial portion of cystic or edematous tissue [17]. Guan and Lin et al. have demonstrated that cervical cancer had a positive skewness while the skewness of normal cervix was much lower and close to 0 [5, 7]. Effective treatment results in a gradual disappearance of tumor’s high cellularity and a more homogeneous distribution of ADC values within the tumor, which makes skewness decrease as we have observed in this study.

As for kurtosis, we found that its change was completely consistent with the change of skewness. In our study and King et al.’s study, kurtosis showed a significant decrease since the early stage of treatment [11], while Kyriazi and Tyagi et al. both found that kurtosis first increased and then decreased at the later stage of therapy [10, 16]. Higher kurtosis indicates a sharper peak and wider tails of the distribution of ADC values. Guan et al. have demonstrated that kurtosis of normal cervix was significantly lower than that of cervical cancer because normal tissues were relatively more homogeneous [5]. After receiving effective therapy, ADC values of the tumor tended to be evenly distributed across the range like normal tissues, which made kurtosis go down following therapy. Studies of kurtosis on cervical cancer are still limited. Downey and Lin et al. both demonstrated that kurtosis showed no value for cervical cancer classification or grading while it had a certain value for differentiating cervical cancer from normal cervix [4, 7]. Our study firstly proved that kurtosis had potential as a prognostic biomarker for cervical cancer.

Our study also firstly investigated changes of entropy during CCRT in cervical cancer and found that first-order entropy showed a stable decline after the end of 2nd week of CCRT. Several studies found that malignant tumors had greater first-order entropy compared to benign tissues for they were more heterogeneous on cellular morphological level [18, 19]. All responders showed decreasing first-order entropy in our study, indicating that cervical cancer cell morphological heterogeneity was steadily decreasing following effective CCRT.

Second-order entropy is a texture based statistical measure of the randomness in an ADC histogram which not only reflects distribution of ADC values but also covers the spatial information, thus takes more advantages in analyzing tumor microstructural heterogeneity than first-order entropy [20]. Our in-house software calculated 12 s-order entropies deriving from different directions as well as their averaged value. Changes of those second-order entropies proved the same as that of the first-order entropy, indicating a steady decline in cervical cancer microstructural heterogeneity following effective CCRT. As a main part of texture analysis, applications of second-order entropy on tumor have grown in recent years. Ryu et al. reported that second-order entropies based on the entire tumor volume could be useful for evaluating glioma grade and their diagnostic accuracy was significantly higher than that of the 5th percentile [8]. In 2013, Foroutan et al. first applied second-order entropy on tumor prognosis and demonstrated that second-order entropy was able to predict treatment response following cancer therapy at earlier time points than tumor volume changed [14].

To sum up, skewness, kurtosis and entropy all kept decreasing, indicating that the distribution of ADC values became less heterogeneous following CCRT, along with a good response to therapy. Visually, those changes reflected on the averaged ADC histogram which gradually moved toward the right and turned into a more symmetrical shape with conspicuous descending peak following effective therapy.

s-sD_av_ represents width of the waist of ADC histogram. In this study, s-sD_av_’s increase occurred at a very late stage of CCRT. Therefore, it may not be a suitable indicator for early detection of cervical cancer treatment response. To the best of our knowledge, s-sDav has been reported only on the diagnosis of hypoxic ischemia encephalopathy [21] and it has never been applied in cancer research previously.

There was only one study on width related to tumor therapy reported by Nishiguchi et al. who found that width of ADC histogram in meningioma increased significantly after embolization therapy [22]. While in our study, width of cervical cancer didn’t show any significant change during CCRT.

Our study firstly found standard deviation of cervical cancer remained stable during CCRT. Changes of standard deviation are closely related to tumor pathological changes in the process of anti-cancer therapy. After effective treatment, if solid components gradually turn into tissues with lower cellularity, ADC values will distribute more homogeneously, which results in a lower standard deviation. However, if there are fibrosis or residual solid components existing after treatment, standard deviation may increase instead. Thus, standard deviation may not be stable and reliable enough to serve as a prognostic biomarker for cervical cancer.

Our study had several limitations. Firstly, the sample size was relatively small. Only 32 patients were enrolled and all of them were responders (27 as CR and 5 as PR), lacking nonresponders who were classified as SD or PD as a control. Due to limited cases of PR, we failed to compare the differences of ADC histogram parameters between CR and PR. It has been reported that ADC values could predict the outcome after CCRT in advanced cervical cancers [2, 23, 24]. For example, Liu et al. [23] and Kuang et al. [2] both demonstrated that the ADC change percentage of CR group at early follow-up time was greater than that of PR group. However, there are still some inconsistencies. Liu et al. [24] reported that baseline ADC value of CR group was significantly lower than that of PR, while Kuang et al. [2] failed to detect any significant difference of baseline ADC values among CR, PR and SD groups. Further studies with a larger number of patients are needed. Secondly, there may be a selection bias due to the inclusion of only squamous cell carcinomas in our study. Thirdly, we did not use the pathologic response as reference standard, and we could not correlate changes of certain parameters with pathological findings. Fourthly, entropy was regarded as one of histogram shape related parameters though it was not a pure morphological parameter. Fifthly, tumor response was only based on tumor diameter measurement immediately after CCRT completion, lacking long-term follow up outcomes.

## Conclusions

In conclusion, our study showed that the whole-lesion ADC histogram shape analysis hold potential for the early detection of cervical cancer response to CCRT, and may provide an opportunity for clinicians to adjust therapeutic strategies such as radiation dose in time to develop a more individualized treatment.

## Abbreviations

ADC: Apparent diffusion coefficient
CCRT: Concurrent chemo-radiotherapy
CR: Complete response
CT: Computed tomography
DW: Diffusion weighted
EBRT: External beam radiation therapy
HNSCC: Head and neck squamous carcinoma
ICBT: Intracavitary brachytherapy
MR: Magnetic resonance
PD: Progressive disease
PR: Partial response
RECIST: Response evaluation criteria in solid tumors
ROIs: Regions of interest
RT: Radiotherapy
SD: Stable disease
